# Intensity dependent effect of cognitive training on motor cortical plasticity and cognitive performance in humans

**DOI:** 10.1007/s00221-020-05933-5

**Published:** 2020-10-06

**Authors:** Christina Berns, Wanja Brüchle, Sebastian Scho, Jessica Schneefeld, Udo Schneider, Karin Rosenkranz

**Affiliations:** Ruhr- University of Bochum, Medical Faculty, University Clinic for Psychiatry and Psychotherapy, Campus East-Westphalia, Virchowstraße 65, 32312 Luebbecke, Germany

**Keywords:** Transcranial magnetic stimulation, Brain plasticity, Paired associative stimulation, Cognition, Human, Neurorehabilitation

## Abstract

Intervention-induced neuroplastic changes within the motor or cognitive system have been shown in the human brain. While cognitive and motor brain areas are densely interconnected, it is unclear whether this interconnectivity allows for a shared susceptibility to neuroplastic changes. Using the preparation for a theoretical exam as training intervention that primarily engages the cognitive system, we tested the hypothesis whether neuroplasticity acts across interconnected brain areas by investigating the effect on excitability and synaptic plasticity in the motor cortex. 39 healthy students (23 female) underwent 4 weeks of cognitive training while revision time, physical activity, concentration, fatigue, sleep quality and stress were monitored. Before and after cognitive training, cognitive performance was evaluated, as well as motor excitability using transcranial magnetic stimulation and long-term-potentiation-like (LTP-like) plasticity using paired-associative-stimulation (PAS). Cognitive training ranged individually from 1 to 7 h/day and enhanced attention and verbal working memory. While motor excitability did not change, LTP-like plasticity increased in an intensity-depending manner: the longer the daily revision time, the smaller the increase of neuroplasticity, and vice versa. This effect was not influenced by physical activity, concentration, fatigue, sleep quality or stress. Motor cortical plasticity is strengthened by a behavioural intervention that primarily engages cognitive brain areas. We suggest that this effect is due to an enhanced susceptibility to LTP-like plasticity, probably induced by heterosynaptic activity that modulates postsynaptic excitability in motorcortical neurones. The smaller increase of PAS efficiency with higher cognitive training intensity suggests a mechanism that balances and stabilises the susceptibility for synaptic potentiation.

## Introduction

The human brain is plastic and optimises its functions in adaption to challenges. Several studies in humans have focused on the brain’s changes in the context of motor learning and described short- and long-term functional and structural changes (Sale et al. 2017; Ziemann et al. 2004; Gaser and Schlaug 2003; Rosenkranz et al. 2007a, b). Other studies explored the effect of cognitive training and have shown structural brain adaptation (Takeuchi et al. 2014; Ceccarelli et al. 2009) e.g. in London taxi drivers (Maguire et al. 2000) and medical students (Draganski et al. 2006).

While these studies showed neuroplasticity within the motor or cognitive systems, fewer studies investigated training-induced effects across different brain systems, e.g. of physical exercise inducing structural changes in the cognitive network and improving cognitive performance (e.g. Wagner et al. 2015; Erickson et al. 2011; Li et al. 2017; Hötting and Röder 2013; Valkenborghs et al. 2019).

The different areas of the brain are densely interconnected: especially the motor cortex represents an important node and processes information from various inputs (Tomasi and Volkow 2011). Several key structures of the cognitive network, such as the dorsolateral prefrontal cortex (Takeuchi et al. 2014), the posterior parietal cortex (Draganski and May 2008), as well as frontal areas (Ceccarelli et al. 2009) have been shown to be connected to the motor cortex, as several double-pulse TMS studies (Hasan et al. 2013; Koch et al. 2007; Davare et al. 2008; Civardi et al. 2001) as well as cortico-cortical paired associative stimulation studies (Chao et al. 2015; Veniero et al. 2013; Koch et al. 2013; Kohl et al. 2019) that test and modulate these interconnections have confirmed.

Here we investigated whether a specific training that primarily engages selected brain areas induces changes in connected areas that are not primarily engaged in the specific training. We used intensive revision in preparation for a theoretical exam as a model of a cognitive training intervention. Similar to previous studies (Draganski et al. 2006) we recruited healthy university students who envisaged their theoretical end-of-term exams. Our hypotheses were (i) that cognitive training induces a behavioural within-system effect and increases cognitive performance and (ii) that it also acts across systems as such as it influences neural excitability and plasticity in the motor cortex. The cognitive training intervention lasted four weeks, during which the daily amount of time spent revising and of physical activity were carefully monitored. In order to define the intensity of cognitive training, the subjectively perceived levels of concentration, of fatigue and of stress, as well as the quality of sleep were obtained in addition to daily revision time. Other potentially influencing factors, (e.g. intensive hand training, medication, alcohol or drug intake) were monitored as well. Similar to the intensity-dependent effect of motor training on motor cortical plasticity (e.g. Rosenkranz et al. 2007a) we predicted the intensity of cognitive training to modulate neuroplastic changes. As physical activity and sport are known modulating factors of neuroplasticity we tested our findings carefully for any potential bias.

## Materials and methods

### Subjects

Thirty-nine healthy students (23 female; 16 male) envisaging end-of-term exams which necessitated several weeks of intensive revisions were recruited. As these were theoretical exams, the students were sedentary while revising and did not engage in rehearsing practical techniques.

All participants gave written informed consent prior to their inclusion in the study, which was approved by the ethics committee of the Ruhr University of Bochum, Medical Faculty, in Bad Oeynhausen/Germany, and conformed to the Declaration of Helsinki.

All participants were students either at the Medical Faculty of the Ruhr University Bochum, Campus East-Westphalia, or at the University of Applied Sciences in Minden. None of the participants engaged in intensive hand motor training (e.g. playing musical instrument or gaming device for > 1 h/week; further details of subjects’ characteristics, see Table 1).

**Table 1:** Subjects characteristics

Age (years ± SEM)	23.67 ± 0.39
Male/female (*N*)	16/23
Right/Left handed (*N*)	35/4
Topic of study (*N*)	
Medicine	21
Nursing	10
Engineering	7
Economy	1

### Neurophysiology

#### Transcranial magnetic stimulation (TMS)

TMS was performed using a Magstim 200 stimulator connected to a figure-of-eight-shaped coil with an internal wing diameter of 7 cm (Magstim Company Ltd, Whitland, UK). The coil was held with the handle pointing backward and laterally 45° to the interhemispheric line to evoke anteriorly directed current in the brain and was optimally positioned to obtain motor-evoked potentials (MEPs) in the abductor pollicis brevis (APB) muscle in the dominant hand. Stimulation intensities are quoted as percentage of maximal stimulator output (mean ± SEM).

#### EMG recording

Surface electromyographic (EMG) recordings in a belly-to-tendon montage were made from the APB and the first dorsal interosseus (FDI) muscles of the dominant hand. The raw signal was amplified and filtered with a bandpass filter of 30 Hz to 1 kHz (Digitimer D360; Welwyn Garden City, UK). Signals were digitized at 2 kHz (CED Power1401, Cambridge Electronic Design, UK) and stored on a laboratory computer for offline analysis. Online EMG was used to control for muscle relaxation during data recording and trials showing voluntary muscle activation were discarded from the analysis (< 1% of trials).

#### Experimental parameters

##### Motor excitability

At the start of each experiment, the active motor threshold (aMT) defined as the minimum intensity needed to evoke a MEP of ≥ 200 µV in 5 of 10 trials in the tonically active APB (approximately 30% of maximal contraction as assessed visually using the online EMG recording) and the stimulus intensity (SI) needed to evoke a MEP of approximately 1 mV peak-to-peak amplitude (SI_1mV_) in the ABP were defined. The input–output relationship of MEP amplitude to stimulus intensity (IOcurve) was measured. Five MEPs each were recorded with 50%, 70%, 80%, 90%, 100% (equal to SI_1mV_), 110%, 120%, 130%, and 150% of SI_1mV_. The mean MEP amplitude per stimulus intensity was calculated for each subject. Furthermore, the steepness of the IOcurve slopes defined as the steepness of the linear regression line through the given data points between 80% and 120% SI_1mV_ (IOslope) were calculated.

##### Short interval intracortical inhibition (SICI)

SICI was measured using two MagStim 200 connected via a BiStim module (Magstim Company Ltd, Whitland, UK). Three different subthreshold conditioning stimulus intensities (70%, 80%, and 90% of aMT) were used to test the input– output relation for SICI (SICI curve; Orth et al. 2003; Rosenkranz et al. 2007b). The conditioning stimulus preceded the suprathreshold test stimulus (intensity set at SI_1mV_) by 3 ms (Kujirai et al. 1993).

Three blocks consisting of 30 trials each were performed. Each block examined one conditioning pulse intensity and consisted of 15 MEPs elicited by the test stimulus alone (test MEPs) and 15 conditioned MEPs presented in pseudorandom order. The peak-to-peak amplitude of the conditioned and test MEPs was measured for each single trial to calculate the mean amplitude and percentage SICI (conditioned MEP: test MEP) for the three different conditioning stimulus intensities. This approach allowed us to measure the level of SICI at a single conditioning intensity as well as the recruitment of SICI (SICI curve) defined as the increase of SICI with increasing intensities of the conditioning stimulus.

##### Plasticity in the motor cortex as assessed by Paired-associative stimulation (PAS)

PAS consisted of 200 electrical stimuli of the median nerve at the wrist of the relaxed dominant hand paired with a single TMS pulse (at SI_1mV_) over the contralateral hand motor cortex with a rate of 0.25 Hz. TMS single pulses were delivered through a figure-of-eight shaped coil (diameter of each wing 70 mm) connected to a Magstim 200 stimulator and was held in the same position as described above. Electrical stimulation of the median nerve was performed at the wrist with a standard stimulation block (cathode proximal) connected to a Digitimer DS7A stimulator (Digitimer, UK) using square-wave pulses (duration 0.2 ms) at an intensity of three times the perceptual threshold. The electrical stimuli preceded the TMS pulses by 25 ms (PAS25). PAS25 has been shown previously to induce a long-lasting MEP increase (Stefan 2000; Stefan et al. 2002; Ziemann et al. 2004). To keep their attention focused on their hand, subjects were instructed to look at their stimulated hand and count the peripheral electrical stimuli they perceived (Stefan et al. 2004). During PAS, the MEPs evoked in the APB and FDI were displayed on-line on the computer screen to control for the correct coil position and stored for off-line analysis.

Before and 10 min after the end of the PAS-intervention 20 MEPs elicited using SI_1mV_ were recorded and their mean amplitude was calculated. The effect of PAS was defined in each subject as change of the MEP amplitude in the APB (MEP after PAS/MEP before PAS; in percent).

### Cognition

A PC-based test battery (Vienna test system, Schuhfried^®^, Austria) was used to measure different aspects of cognitive performance. The work performance series required subjects to perform additions and subtractions of single-digit numbers as fast and accurate as possible for 7 min (Arnold 1975; Schuhfried 1986) and thus assessed the performance speed and accuracy within an attention task. Executive functions were tested by using the Response Inhibition (RI) and Stroop Interference tests (STROOP). The RI consisted of a GO/NoGo reaction time task (Kaiser et al. 2005, 2010). The STROOP (colour/word interference) gave information about the subjects’ ability to control cognitive interference (Stroop 1935; Schuhfried 1999).

Memory performance was tested with the N-back verbal (NBV) and the California verbal learning test (CVLT). The NBV (2-back paradigm) required subjects to recognise consonants presented two places back out of a series of successively presentedconsonants (Schellig and Schuri 2009). The CVLT tested episodic verbal learning and memory and assessed encoding, recall and recognition, as well as subjects’ learning strategies (e.g. semantic or serial clustering) (Niemann et al. 2014).

### Monitoring of revision habits and daily activities

#### Pre-structured diary

During the cognitive training subjects were asked to document aspects of their daily activities that were relevant for the study in a pre-structured diary. These included the amount of revision (time/day), the amount of physical exercise/activity (time/day). The latter included not only intentional sportive activities, but also other forms of physical activities like cycling to campus, going shopping etc..

#### Questionnaire

In order to complement and extend the information gathered in the pre-structured diary, a questionnaire was used to inquire after daily activities and other relevant aspects to the study. This also ensured the retrieval of this important information in case the subjects did not complete the diary satisfactorily. Subjects were given this questionnaire at the end of the revision period and were prompted (i) to estimate their daily amount of revision time given in time brackets (< 3 h, 3–5 h, 5–7 h or > 7 h), (ii) to report the amount of sport and other physical activities (e.g. cycling to campus) performed per day (hours/day) during the four weeks of cognitive training and also regarding the month before that, in order to get an estimation of their regular level of physical activity; (iii) to self-assess the subjectively perceived level of stress during the four weeks of revision time as low, moderate or high, and (iv) to assess their subjectively perceived level of concentration, level of fatigue and quality of sleep during revising on visual-analog scales (VAS) ranging from 0 to 6. Other aspects included were: playing of a musical instrument (if yes, amount of time/week), use of gaming devices (if yes, amount of time/week); movement during revising (e.g. revising in other than sedentary positions; revising while walking around), medication (especially centrally active drugs) and intake of alcohol and/or drugs.

### Experimental design and statistic analysis

#### Experimental protocol (Fig. 1)

**Fig. 1:**
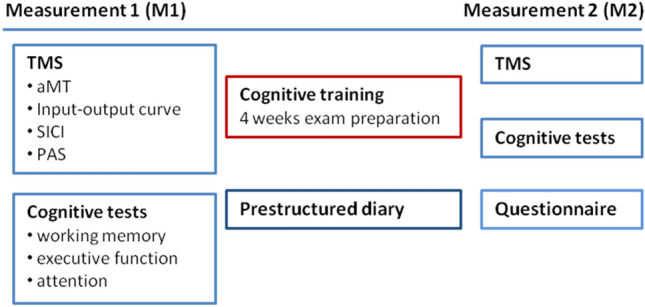
Experimental protocol. Before (M1) and after (M2) the cognitive training, subjects underwent the TMS and cognitive tests. During the cognitive training, subjects were asked to document the amount of time spent revising as well as performing physical activity/sports per day. At the end of the cognitive training, a questionnaire was presented that collected information on revision and physical activity/sport habits, as well as on the subjectively perceived level of stress. Investigators performing the TMS and cognitive tests after cognitive training (M2) were blinded towards the results of the pre-structured diary and the questionnaire

Figure 1 shows the experimental protocol. The cognitive training consisted of revising for the individuals’ exams and lasted four weeks (± 2 days). During this exam preparation period, subjects documented their daily activities in the pre-structured diary; particularly the time spent revising and exercising per day. At the end of the cognitive training the questionnaire was performed (see above *Monitoring of revision habits and daily activities*).

Before the students started revising (measurement 1, M1) and within two days after they underwent their exam (measurement 2, M2), the above mentioned parameters of neuronal excitability (aMT, IOcurve, SICI) and plasticity (PAS-induced plasticity) were measured and the cognitive tests were performed (parallel test versions were used for M2). The experimental parts (neurophysiology and cognition) were always performed in the same order for the individual subject and at the same time of day (within a time frame of 9am to 3 pm). Importantly, the data of questionnaires and diaries of the individual subject were not yet analysed when the neurophysiological and cognitive tests for M2 were undertaken. Thus, the investigators were blinded towards the individual subject’s amount of daily revision time, physical activity and levels of concentration and fatigue, sleep quality and stress level when performing the experiments.

### Data analysis and statistics

All data was tested for normal distribution by use of the Kolmogorov–Smirnov test. In case of not normally distributed data, non-parametric tests were used. All ANOVAs were tested for sphericity using Mauchly’s test. In case of significant sphericity, Greenhouse–Geisser corrections were performed. Effect sizes (η^2^; r) were calculated for significant interactions. All data are given as mean ± SEM. Significance levels for the statistical tests are set to *p *≤ 0.05 (unless otherwise stated).

The TMS parameters (aMT and SI1 mV) of M1 and M2 were tested by paired *t* tests. The *IOcurve* and *SICI* data were analyzed using ANOVA with the within group factors “stimulus intensity” (IOcurve) or “conditioning pulse intensity” (SICI) and “cognitive training,” which refers to the two experimental conditions given by measuring before (M1) and after (M2) cognitive training. The MEPs measured in M1 and M2 before PAS were compared by means of paired t-tests in order to control for correct adjustment of MEP size to 1mV peak-to-peak amplitude. ANOVAs were performed on the raw data of MEPs with the factors “cognition training” and “MEP amplitude before/after PAS.” For further analysis, the MEP raw data were normalized and expressed as percentage of MEPs (MEPs after PAS: MEPs before PAS; PASeffect).

The information given in the diary and pre-structured questionnaire on the time spent with sport and physical activity per day was summarised to give an average per week (hours/week).

To examine the influence of revision time, physical activity/sport, subjectively perceived stress, and the level of concentration, fatigue and sleep quality (assessed by VAS) on the neurophysiological data, further ANOVAs were performed on the IOcurve and IOslope data, the SICI data and the PASeffect data using “cognitive training” as within group factor and “revision time”, “physical activity and sport”, “perceived stress level”, “VAS:concentration”, “VAS:fatigue” or “VAS:sleep” as between group factors. Post hoc tests were performed when necessary.

The raw data of cognitive tests was transformed into T scores (mean = 50; SD = 10); with T-scores > 50 indicating higher, and T-scores < 50 indicating lower performance in comparison to a representative population (matched for age, sex and level of education) as given by the Vienna Test System (Schufried ^®^, Austria). Similar to the analysis of the neurophysiological data, ANOVAs were performed with the factors “cognitive training” as within group factor, “revision time”, “physical activity and sport”, “perceived stress level” or one of the three “VAS” parameters (concentration, fatigue, sleep quality) as between group factors. Post hoc tests were performed when necessary. Furthermore, data obtained before and after the cognitive training was directly compared using either paired-samples t-tests or Wilcoxon tests and their significance levels adjusted to correct for multiple comparisons (Bonferroni).

Correlations between neurophysiological or cognitive data and revision time, physical activity/sport, subjectively perceived level of stress, of concentration, of fatigue or of sleep quality, as well as between neurophysiological and cognitive data were calculated and significant results are reported giving Pearson’s r for normal distributed and Kendall’s tau for non-normal distributed data.

## Results

None of the subjects experienced any discomfort during the experiments or any side effects of TMS testing. There were no significant effects of sex, age, topic of study, order of experiments (neurophysiology or cognition first) or time of day (morning or afternoon) on any of the neurophysiological or cognitive parameters (One-way ANOVAs; n.s.).

### Monitoring of revision habits and daily activities

The subjects readily completed their pre-structured diaries during the revision period and gave detailed information on their daily activities. None of the subjects played a musical instrument or used a gaming device excessively (< 1 h/week) prior to or during the revision time. None of the subjects took any centrally active medication. The average consumption of alcohol was 0.62 ± 0.16 units/week; four subjects reported occasional consumption of cannabis (in each case < 1 g/week). All subjects reported to have been sedentary during revision, that is, they were revising while sitting on a chair at a desk.

#### Revision and physical exercise habits

Table 2a shows the amount of time spent revising per day and Table 3 shows the type of sport subjects engaged in and the amount of time spent performing sport and physical activity per week explored by use of the pre-structured diary and the questionnaire.

**Table 2:** Daily revision time (hours/day) and stress level

	Mean	SEM
(A) Revision time		
Diary (h/day)	2.93	0.25
Questionnaire (h/day)	3.85	0.28

	*N*	% of total *N*
Questionnaire		
< 3 h/day	14	35.90
3–5 h/day	16	41.03
5–7 h/day	7	17.95
> 7 h/day	2	5.13

	*N*	% of total *N*
(B) STRESS (Questionnaire)		
Low	8	20.51
Moderate	18	46.15
High	13	33.33

**A** The daily revision time as given by the pre-structured diary and the questionnaire were significantly different (Wilcoxon; *p* < 0.001); however, both data sets were correlated (Kendall’s tau = 0.55; *p* < 0.001) indicating a systematic variation. **B** Subjects assessed their subjectively perceived level of stress as being low, moderate of high in the questionnaire. The number of subjects and the percentage of total population are given

**Table 3:** Sport and physical activity (hours/week)

Type of sport	*N*	Questionnaire			Diary
		Before: Sport alone	During: Sort alone	During: Sport and physcial activity	During: Sport and physical activity
Endurance	8	1.65 ± 0.34	1.47 ± 0.40	10.52 ± 2.75	9.20 ± 2.73
Strength	4	3.75 ± 1.50	3.75 ± 1.50	7.43 ± 1.23	5.96 ± 1.38
Team sport	4	2.25 ± 0.50	3.00 ± 0.75	7.99 ± 1.75	8.15 ± 2.58
Endurance and strength	7	3.18 ± 0.58	2.57 ± 0.75	9.32 ± 0.88	6.62 ± 0.87
Team sport and endurance	8	3.66 ± 0.62	3.16 ± 0.50	7.88 ± 1.04	7.62 ± 0.69
Team sport and strength	1	2.25	2.25	12.75	12.83
3 or more of the above	2	5.25 ± 0.10	3.75 ± 1.50	10.05 ± 5.70	8.44 ± 2.42
None of the above	5	0	0	4.62 ± 0.63	2.86 ± 0.39
Total	**39**	**2.60 ± 1.93**	**2.35 ± 0.30**	**8.46 ± 0.73**	**7.21 ± 0.72**

The amount of sport and physical activity is given according to the type of sport the subjects engaged in prior to the study. Sport refers to intended sportive activity performed in e.g. a gym, while physical activity refers to additional activity e.g. cycling to campus, going shopping etc. The amount of sport and physical activity during the revision time as measured in the questionnaire and the diary differed significantly (Wilcoxon signed rank test; *p *= 0.009) but were correlated (Kendall’s tau = 0.536; *p* < 0.001). The amount of sport (only) during the revision time and during a four weeks period before exam prepartion was not different (Wilcoxon signed rank test; n.s.)

The subjectively estimated amount of revision time was higher in the questionnaire than in the diary (Wilcoxon; *p* < 0.001), the same applies for the amount of physical activity and sport (Wilcoxon; *p *= 0.009). Measures in the diary and questionnaires were correlated (revision time: Kendall’s tau = 0.55; *p* < 0.001; sport and physical activity: Kendall’s tau = 0.54; *p* < 0.001), indicating that subjects either systematically overestimated their time spent revising and performing sport and physical activity in the retrospectively completed questionnaire, or underestimated them in their diary.

Importantly, the amount of sport performed before and during the exam preparation did not differ significantly (Wilcoxon; n.s.) and was strongly correlated (Kendall’s tau = 0.62; *p* < 0.001), thus indicating that the individuals’ level of sportive activity was constant over the weeks before and during exam preparation.

The time spent revising and performing physical activity were not correlated; neither for the data given by the diary (Pearson’s *r *= − 0.123; *p *= 0.45) nor the questionnaire (Pearson’s *r *= 0.13; *p *= 0.43). Thus, subjects revising for shorter time per day did not have higher levels of physical activity, or vice versa.

#### Questionnaire: Subjectively perceived level of stress

The subjectively perceived level of stress correlated directly with the revision time as measured in the diary (Pearson’s *r *= 0.43; *p* = 0.001) and questionnaire (Pearson’s *r *= 0.35; *p *= 0.015; see Table 2b), but had no influence on or interaction with either neurophysiological or cognitive parameters (One-way ANOVAs; n.s.).

#### Questionnaire: Visual analog scales (VAS) on concentration, fatigue and sleep

**Fig. 2:**
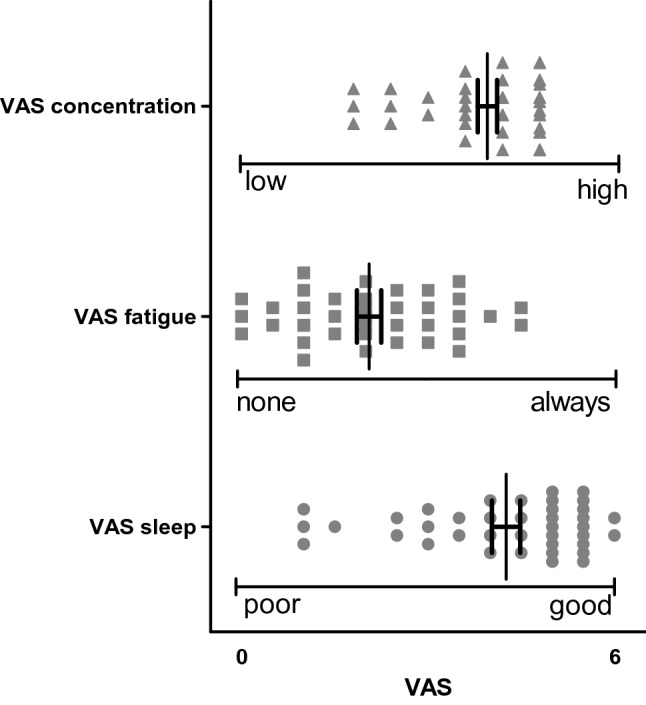
Visual-analog scales (VAS). As part of the questionnaire the VAS measured the level of concentration during revision (0 = low to 6 = high), the occurrence of feelings of fatigue (0 = none to 6 = always) and the quality of sleep (0 = poor to 6 = good). The scatter plot gives the results of the single subjects as well as the mean and SEM. None of the VAS parameters correlated significantly with the revision time given in the diary or in the questionnaire (for all correlations: Kendall’s tau < 0.15 with p < 0.27)

Figure 2 displays the results of the VAS as subjective measures of the level of concentration during revision (0 = low to 6 = high), the occurrence of feelings of fatigue (0 = none to 6 = always) and the quality of sleep (0 = poor to 6 = good). The level of concentration was moderately high (3.94 ± 0.16), the feelings of fatigue occurred infrequently (2.05 ± 0.20) and the quality of sleep was moderately good (4.26 ± 0.23). None of the parameters influenced or interacted with either neurophysiological or cognitive parameters (One-way ANOVAs; n.s.).

#### Correlation between concentration, fatigue and sleep (VAS scales) and revision time

None of the VAS parameters correlated significantly with the revision time given in the diary or in the questionnaire (for all correlations: Kendall’s tau < 0.15 with p > 0.27); thus the amount of time spent revising per day did not influence the subjects’ perception of their level of concentration or of fatigue, or their sleep quality.

### Neurophysiology

The values for aMT were comparable before and after the cognitive training (see Table 4). The SI1mV was slightly lower after cognitive training (*t* test; *p *= 0.043). However, the difference was small (1.74% of maximum stimulator output) and is therefore of minor relevance to the outcome of the experiments involving TMS pulses given at SI_1mV_.

**Table 4:** TMS and PAS parameters

	M1		M2		*p* value
	Mean	SEM	Mean	SEM	
aMT	31.95	0.85	30.59	0.84	0.069
SI_1mV_	50.97	1.44	49.23	1.14	0.043
PAS: sensory threshold (mA)	26.11	1.35	26.95	1.28	0.497
PAS: sensory stimuli counted (N)	197.44	1.86	194.74	4.71	0.443

TMS parameters measured in the APB are given in percentage of maximum stimulator output (± SEM); the sensory threshold measured in the median nerve at the wrist and the number of sensory stimuli counted during application of PAS are given. Results of the statistical analysis (paired t-test) comparing M1 and M2 measures are given

### Motor excitability

#### Input–output curves

**Fig. 3:**
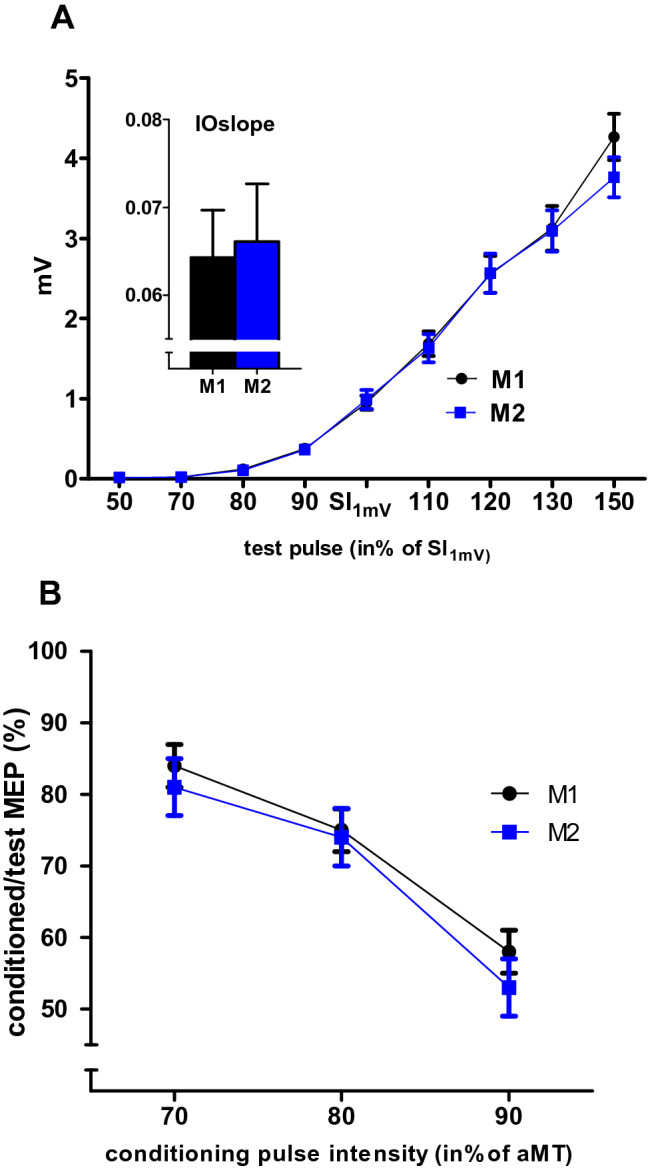
Motor excitability: IOcurves and SICI in the APB. **a** IOcurve: the mean MEP amplitude (in mV ± SEM) as given on the *y*-axis against the stimulus intensity given on the *x*-axis (in percentage of SI_1mV_) measured before (M1; black) and after (M2; blue) the cognitive training. The inset figure displays the IOslope calculated for the approximately linear part of the IOcurve between 80% and 120% SI_1mV_. IOcurves and IOslopes were not different in M1 and M2. **b** SICI obtained with a conditioning pulse intensity of 70%, 80%, and 90% of aMT. The *y*-axis plots the amplitude of the conditioned MEP as the percentage of MEP evoked by the test pulse alone (± SEM). SICI increased with increasing conditioning pulse intensity; however, there was no significant difference between M1 and M2

Figure 3a shows that the IOcurves measured before and after the cognitive training were quite similar. A two-way ANOVA with the within group factors “stimulus intensity” and “cognitive training” revealed a significant interaction (ANOVA F(2.7;102.74) = 69.48; *p* < 0.001), with a strong and significant main effect of “stimulus intensity” (ANOVA; F(1.82; 69.03) = 153.63; *p* < 0.001). The IOslopes calculated on both IOcurves (before and after the cognitive training) were not significantly different (Wilcoxon; *p *= 0.87; ANOVA n.s.).

#### Short-interval intracortical inhibition

Figure 3b shows the results of the SICI protocol, measured with conditioning pulse intensities of 70%, 80%, and 90% of aMT, before and after the cognitive training. SICI increased with stronger conditioning stimuli intensities. The ANOVA with the factors “conditioning pulse intensity” and “cognitive training” showed a significant main effect of “conditioning pulse intensity” (ANOVA, F(1.62;61.54) = 45.17; *p* < 0.001), but no significant interaction or main effect of “cognitive training” (ANOVA; *p *> 0.27).

#### Influence of revision time, physical activity/sport, levels of stress, concentration, fatigue and sleep

Neither the individuals’ revision time per day nor the time spent on sports and physical activity per week had any influence on the IOslope or IOcurve (ANOVA; all p > 0.15) or on SICI (ANOVA all *p* > 0.12). None of the VAS parameters or the level of stress had any significant interaction with IOslope or IOcurve (ANOVA: all *p* < 0.12) or on SICI (ANOVA; all *p* < 0.19).

### Motor plasticity (PAS)

The amplitudes of MEPs measured with SI_1mV_ before the application of the PAS protocol were comparable before (M1) and after (M2) the cognitive training (paired t-test; *p *= 0.73). The sensory threshold of the median nerve stimulation was similar in M1 and M2, as well as the total number of counted peripheral stimuli (see Table 4).

**Fig. 4:**
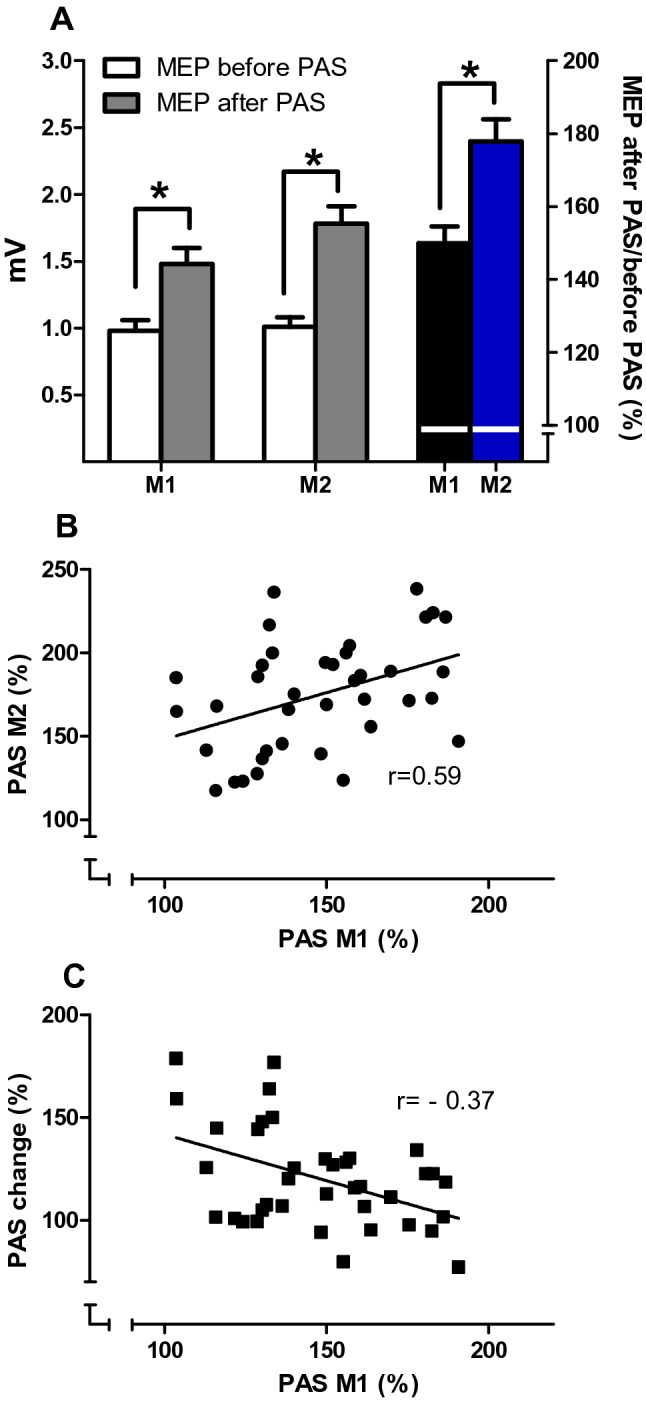
**a** Mean MEP (± SEM) in the APB measured before (white columns) and after (grey columns) PAS at M1 and M2. In M1 and M2 there was a significant increase of MEP amplitude (both paired t-tests; *p* < 0.001). The PAS effect (MEP after/MEP before PAS, in  %; right y-axis) was different in M1 (black column) and M2 (blue column) (paired t-test; *p* < 0.001). (*Marks significant results; paired *t* tests, with *p* < 0.001). **b** shows the correlation of the PAS effects measured at M1 and M2 and **c** the correlation of PAS M1 to PAS change. Both correlations were significant (*p* < 0.018); the coefficients are given in the figure

Figure 4a shows that the PAS25 protocol significantly (paired *t* test; *p* < 0.001) increased the MEP amplitude before (0.98 ± 0.08 mV to 1.48 ± 0.12 mV) and after the cognitive training (1.01 ± 0.07 mV to 1.78 ± 0.13 mV).

However, the PAS effect (mean MEP amplitude after/before PAS, given in  %) was stronger after (177.9 ± 6.1%) than before the cognitive training (149.89 ± 4.55%). An ANOVA with the factors “cognitive training” and “before/after PAS” showed a significant interaction of both factors (ANOVA; F(1;38) = 11.55; *p *= 0.002; η^2^ = 0.23; *r *= 0.48), with a significant main effect of “cognitive training” (ANOVA; F(1;38) = 15.68; *p* < 0.001).

#### Influence of baseline PAS on further measures of PAS

The PAS effect measured before the cognitive training (M1) showed a moderate correlation to PAS effect measured thereafter (M2) (Fig. 4b; Pearson’s *r *= 0.585; *p* < 0.001), thus the stronger PAS was at baseline, the stronger was PAS after cognitive training. The PAS at M1 showed a weak correlation to PAS change (Fig. 4c, Pearson’s *r* = − 0.377; *p* < 0.018) indicating that the baseline level of PAS did not determine the change of PAS effect with cognitive training and that there was no “ceiling effect”.

#### Influence of revision time

**Fig. 5:**
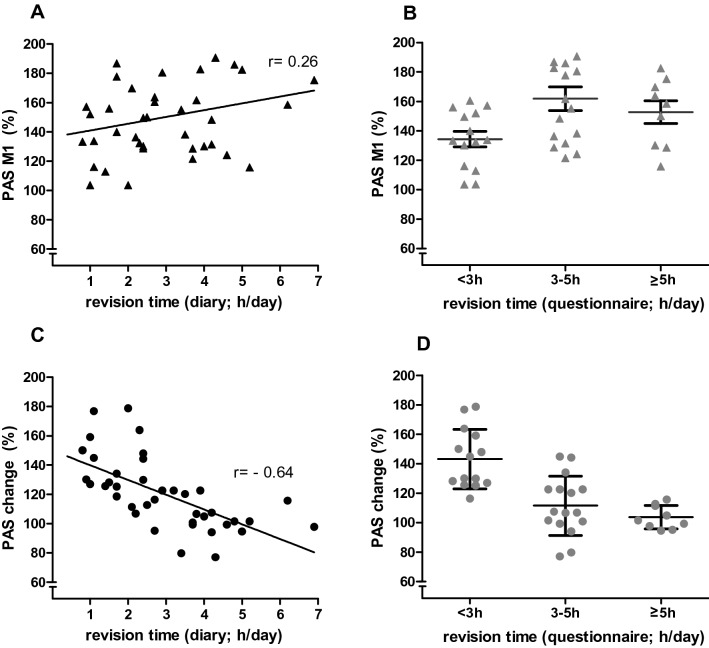
The linear regression between the revision time as given in the diary (h/day; *x*-axis; **a** and **c**) and in the questionnaire (h/day; x-axis; **b** and **d**) with PAS M1 (**a**, **b**) and with PAS change (**c**, **d**; *y*-axis), respectively. The results of the correlation analyses are given in the figures. For the scatter diagrams (**b**, **d**) the subjects who revised ≥ 5 h/day were summarised into one group. The horizontal line within each scatter represents the mean (± SEM; error bars)

There was no significant correlation of the baseline PAS effect (M1) and revision times as given in the diary (Fig. 5a, Pearson’s *r *= 0.26; *p *= 0.11) or the questionnaire (Fig. 5b, Kendall’s tau = 0.24; *p *= 0.062). The change of PAS effect (PAS change = PAS effect M2: PAS effect M1, in  %) was significantly and inversely correlated to the revision time as given in the diary (Fig. 5c; Pearson’s *r *= − 0.64; *p* < 0.001) and in the questionnaire (Fig. 5d; Kendall’s tau = − 0.57; *p* < 0.001). This indicates that the more intensive the students revised (in h/day) the smaller their PAS effect increased after the cognitive training. This finding was confirmed by ANOVAs calculated on the PAS effect data using “cognitive training” and “revision time” as factors: using revision time as given in the questionnaire as between group factor, there was a significant two way interaction (ANOVA; F(3;35) = 5.742; *p *= 0.003; η^2^ = 0.72, *r *= 0.85), with “revision time (questionnaire)” having a significant main effect (F(1;35) = 9.29;*p *= 0.004). Similarly, using revision time as given in the diary as covariate, there was a significant interaction (ANOVA; F(1;37) = 25.63; *p* < 0.001; η^2^ = 0.09; *r *= 0.3), with a significant main effect of “cognitive training” (F1;37) = 59.45; < 0.001).

#### Influence of sport and physical activity, of the levels of stress, concentration, fatigue or sleep

In contrast to revision time, neither the amount of sport alone or sport and physical activity nor the type of sport performed had an effect on PAS measured before (M1) or after the cognitive training (M2), or on the PASchange. Correlation analyses and ANOVAs did not show any significant results. Furthermore, there were no significant interactions of the level of stress or any of the VAS parameters (level of concentration, fatigue or sleep) as between group factors with the PAS effect (ANOVA; all *p* < 0.122), but all of them showed a significant main effect of the within group factor “cognitive training” (ANOVA; *p* < 0.001). Furthermore, there was no significant correlation between PAS and any of the VAS parameters or the level of stress.

### Cognition

Table 5 shows the main results (T scores ± SEM) of cognitive tests performed before and after cognitive training (including statistics). Several parameters indicated a trend towards an improvement of performance; however, after correcting for multiple comparisons there were only few differences that could be considered as significant (Table 5; *p* were adjusted to ≤ 0.01, given in bold).

**Table 5:** Results of cognitive tests given (T scores)

	M1		M2		Statistics
	Mean	SEM	Mean	SEM	(*p * value; paired samples *t* test; *Wilcoxon signed rank test)
Attention					
Work performance series					
Total items worked	50.69	1.32	54.74	1.32	*p < 0.001
Errors	43.08	1.70	42.33	1.61	*p = 0.63
Increase of items worked	50.21	1.53	47.85	1.71	*p = 0.21
Executive function					
STROOP test					
Reading interference	51.15	1.42	51.23	1.28	*p *= 0.945
Naming interference	52.51	1.52	53.85	1.39	*p *= 0.233
Baseline condition: speed reading words	60.38	1.58	65.15	1.59	*p* < 0.001
Baseline condition: speed naming colours	59.69	1.40	63.67	1.56	*p* < 0.001
Interference condition: speed reading words	58.44	1.66	61.79	1.68	*p *= 0.001
Interference condition: speed naming colours	59.33	1.74	63.44	1.76	*p* < 0.001
Response inhibition					
Comission errors	49.92	1.21	52.31	1.39	*p = 0.082
Omission errors	48.64	1.00	48.59	1.20	*p= 0.746
Sensitivity index	50.31	1.21	52.18	1.57	*p* = 0.237
Mean time “correct answers”	51.77	1.67	51.69	1.72	*p* = 0.939
Working Memory					
N-back verbal					
Correct answers	65.36	2.73	70.23	2.39	*p = 0.012
Omissions	59.36	2.45	64.90	2.42	*p = 0.011
Errors	56.46	1.16	59.00	1.09	*p = 0.038
Mean time “correct answers”	57.54	1.62	61.67	1.31	*p = 0.011
California verbal learning test (CVLT)					
Learning sum	45.21	1.48	43.56	1.45	*p* = 0.181
Delayed free recall I	48.18	1.28	50.33	1.66	*p* = 0.061
Delayed free recall II	46.51	1.64	50.33	1.48	*p* = 0.007
Cued recall I	46.92	1.35	46.69	1.73	*p* = 0.825
Cued recall II	47.31	1.33	46.74	1.58	*p* = 0.601
Semantic clustering	35.85	0.21	31.03	0.26	*p < 0.001
Serial clustering	56.21	1.51	54.97	2.26	*p* = 0.578
Learning curve	46.92	1.67	50.18	1.80	*p = 0.094

In the work performance series the significant increase of completed items with constant error rate indicates an improvement of attention. Likewise, the results of the STROOP test indicate a more general increase of working speed: the speed of word reading and colour naming increased similarly under baseline and interference conditions, while the colour/word interference as a marker of executive functions did not change.

Working memory performance improved with cognitive training as shown by the NBV test: subjects gave more correct answers in shorter time and showed fewer errors.

In the CVLT, only the parameter semantic clustering showed a significant change in a direct before-after comparison. Since other main parameters, e.g. learning sum and learning curve, are unchanged, this finding is of minor relevance.

There were no other significant findings or interactions of cognitive performance parameters with other parameters.

## Discussion

This study explored the effect of a period of cognitive training on cognitive performance and on the neural excitability and plasticity in the motor cortex. The main findings are that cognitive training (i) enhances attention and verbal working memory, ii) does not change neuronal excitability, but enhances neuroplasticity in the motor cortex in an intensity-depending manner: the more intensive the cognitive training, the smaller the increase of neuroplasticity; and that (iii) these effects are neither influenced by physical activity or sport, nor by the subjectively perceived levels of concentration, fatigue, stress, or sleep quality.

We chose the revision for a theoretical exam –precluding rehearsal of practical techniques—as a model of cognitive training since it does not engage motor areas per se, thus any modulation of motor excitability or plasticity is probably induced indirectly, that means across systems.

Revising for end-of-term exams required the subjects to memorise a high amount of abstract information over a defined period of time and to recall it on the day of the exam, which strongly resembles working memory training. The latter has been shown not only to lead to behavioural improvements in working memory- and attention control-dependent tasks, but also to increase plasticity in an intraparietal-prefrontal network that is common for working memory and control of attention (Klingberg 2010).

The subjects in our study were young and healthy university students with a high level of cognitive performance at baseline. While executive functions were unchanged, there was a behavioural gain in verbal working memory performance (N-back verbal test) and in attention (work performance series) after the cognitive training. As the latter test had no parallel version, the slight improvement could be due to the subjects being familiar with the test. All in all, the changes in cognition were subtle, and four weeks might have been too short an observation period to induce stronger alterations, especially since the subjects’ baseline performance was already slightly above average.

As expected, the students’ daily amount of time spent revising in preparation for their exams was individually quite different and ranged between 1-7 h/day, as given by their self-report in the diary. Interestingly, the PAS effect increased stronger in those students, whose daily revision time was shorter, especially shorter than three hours/day. As the amount of revision varied across medical and nursing students, these differences cannot be explained by the topic of study. Apart from the mere duration in hours/day, the efficiency of revision is likely to be important, which –in turn – is influenced by factors like the levels of concentration, of fatigue and the quality of sleep. Our subjects were prompted to report on these aspects using self-assessment scales (VAS). It is difficult to obtain objective measures of these factors, and self-assessment has its limitations as it is –by nature—highly subjective. However, it might not be the “objective” level of e.g. fatigue, but how the subjects are subjectively affected by e.g. feeling tired, which is the operative factor that influences the efficiency of revision. For this reason we do think that the results of VAS give reasonably valid estimations of the level of concentration, of fatigue and the quality of sleep, which all together had no influence on any neurophysiological parameter, particularly not on the change of the PAS effect with cognitive training. In addition, they were not correlated to the revision times reported by the subjects in the diary and the questionnaire, indicating that revising for a shorter time is not necessarily associated with higher concentration levels (or vice versa), and that revising for a longer times does not go along with higher levels of fatigue (or vice versa).

Furthermore, the individuals’ amount of time spent with physical activity/sport and with revision were not correlated; thus, the subjects with shorter revision times per day did not spent more time with physical activity and sport. The latter are known to influence neuroplasticity (Ridding and Ziemann 2010; Rosenkranz et al. 2007a, b; Mellow et al. 2020; Huang 2016). Here, the PASeffect changes induced by cognitive training were not influenced by the individuals’ level of sport and physical activity. The study population consisted of young and healthy students with an active lifestyle who did not alter their physical activity habits during the exam preparation time. It is therefore likely, that their baseline level of neuroplasticity was already adapted to their habitual level of physical activity at the start of the study and that physical activity/sport performed during the revision period did not contribute to PAS changes.

Taken together, it is very likely that the amount of revision time per day is the modulating factor of neuroplasticity in the motor cortex.

We did not include a control group in our study. As the individuals’ revision time covered such a wide range, and especially included short periods of one hour per day, including a control group of subjects would have required to recruit students who revise even less than that or not at all. Several studies have reported that the response to PAS, and also to other non-invasive brain stimulation protocols, shows inter- and intraindividual variability in healthy subjects (Huang et al. 2017; Guerra et al. 2017a, b; López-Alonso et al. 2014; Müller-Dahlhaus et al. 2008). This is due to several factors: while age, gender and genetics are factors that cause mainly inter-individual variability, time of day and physical activity habits may influence subjects’ response from session to session and contribute to both inter- and intra-individual variability (Huang et al. 2017). By recruiting subjects within a relatively narrow age range (20-31 years), monitoring their physical activity habits (including muscle activity prior to PAS) and performing the TMS measures of M1 and M2 at the same time of day, we intended to reduce these factors as much as possible (Guerra et al. 2017a; Huang et al. 2017). Using a comparable subject population, the effects of PAS applied subsequently within an interval of several weeks have been shown to be comparable (Müller-Dahlhaus et al. 2007).

All our subjects responded to the PAS 25 protocol by increasing the mean MEP amplitude before (lowest PAS effect was 103.70% at M1) and after cognitive training (lowest PAS effect 117.53% at M2). This high rate of facilitatory PAS response might be due to the subjects’ age and physical activity level, e.g. cycling to campus. The correlation of PAS M1 and M2 was moderately strong (Pearson’s *r *= 0.59; *p* < 0.001; see Fig. 4b) showing that in most subjects (31 of 39) PAS M2 was higher than PAS M1; indicating that the individuals’ measures of PAS were not stochastically but directionally modulated. Importantly, there was only a weak correlation of the baseline PAS effect (M1) and PAS change (Pearson’s *r *= − 0.38; *p *= 0.018; see Fig. 4c). Thus, the individuals’ baseline PAS did not determine the amount of PAS change after cognitive training, which makes a “ceiling effect” unlikely. PAS change – but not the baseline PAS effect (M1)—was correlated with revision time, which shows that—irrespective of baseline PAS—cognitive training induced changes to the PAS effect that were dependent only on the amount of revision time. As measures of cortical excitability were unchanged, alterations in neuronal or interneuronal recruitment are unlikely to contribute to this effect.

Therefore, cognitive training is likely to have had an influence on the efficiency of the PAS protocol to induce LTP-like plasticity in the motor cortex.

By which mechanism might a behavioural cognitive intervention influence synaptic plasticity in the motor cortex? We suggest that cognitive training activates synaptic connections to motor cortical neurones which influence postsynaptic excitability—potentially by shifting the postsynaptic membrane potential closer to firing threshold—and consequently facilitate the induction of LTP-like plasticity (Chistiakova et al. 2014; Huang et al. 2018; Ibata et al. 2008).

Then, how could the intensity-dependent effect of cognitive training be explained? Simply longer might not mean better (Gamboa et al. 2010). Similar to studies using non-invasive brain stimulation which showed that applying two LTP-like plasticity inducing PAS protocols successively (within 30 min) reduces the PAS effect (Müller-Dahlhaus et al. 2007) and that prolonging intermittend theta-burst stimulation (iTBS; Gamboa et al. 2010) even reverses the facilitatory effects on MEPs, prolonging the daily sessions of cognitive training might have induced an “overtraining effect”. With revision times getting longer the training-free intervals are getting shorter. Consequently, the effect of a training session might interfere stronger with the neuroplastic changes induced by prior sessions, in accordance with mechanisms of homeostatic plasticity. Yet here the cognitive training is likely to activate synaptic connections to the motor cortex that are different from those engaged in PAS, which points to a heterosynaptic mechanism. The latter stabilises neural excitability by balancing synaptic weights within a neurone (Chistiakova et al. 2014; Ni et al. 2014; Stanton 1996). The smaller increase of PAS change in students with long revision times might indicate that the induction of LTP-like plasticity gets balanced in order to stabilise neuronal excitability and synaptic modifiability within a certain range.

## Conclusion

We have shown that a behavioural intervention with cognitive training which primarily engages the cognitive network in the human brain influences synaptic plasticity in the motor cortex and that this effect is influenced by the intensity of the training. This cross-system neuroplasticity is likely to act through the dense interconnections between cognitive and motor areas. While this intensity-dependent effect of cognitive training on PAS-induced plasticity is of importance with regard to clinical application of cognitive training interventions, e.g. against age-related cognitive decline or in neurorehabilitation (Antonenko et al. 2018; Antonenko et al. 2019; Bherer 2015), it is at the same time not straight-forward to interpret and necessitates further investigations.

## Data Availability

All data and materials as well as software applications or custom code support the published claims and comply with standard in the research field.
